# Measuring ethical behavior with AI and natural language processing to assess business success

**DOI:** 10.1038/s41598-022-14101-4

**Published:** 2022-06-17

**Authors:** Peter Gloor, Andrea Fronzetti Colladon, Francesca Grippa

**Affiliations:** 1 MIT Center for Collective Intelligence, 245 First Street, Cambridge, MA 02142 USA; 2 Department of Engineering, University of Perugia, Via G. Duranti 93, 06125 Perugia, Italy; 3 Northeastern University, 360 Huntington Avenue, Boston, MA 02115 USA

**Keywords:** Information technology, Computer science, Human behaviour, Complex networks

## Abstract

Everybody claims to be ethical. However, there is a huge difference between declaring ethical behavior and living up to high ethical standards. In this paper, we demonstrate that “hidden honest signals” in the language and the use of “small words” can show true moral values and behavior of individuals and organizations and that this ethical behavior is correlated to real-world success; however not always in the direction we might expect. Leveraging the latest advances of AI in natural language processing (NLP), we construct three different “tribes” of ethical, moral, and non-ethical people, based on Twitter feeds of people of known high and low ethics and morals: fair and modest collaborators codified as ethical “bees”; hard-working competitive workers as moral “ants”; and selfish, arrogant people as non-ethical “leeches”. Results from three studies involving a total of 49 workgroups and 281 individuals within three different industries (healthcare, business consulting, and higher education) confirm the validity of our model. Associating membership in ethical or unethical tribes with performance, we find that being ethical correlates positively or negatively with success depending on the context.

## The importance of ethics and morality for business

German army sergeant Anton Schmid was executed as a traitor by the German army for saving 300 Jews by shielding them from the Ponary massacre. While Schmid was recognized by Israel right after the Second World War, Schmid’s widow was refused a pension after the war, and her windows were smashed by the neighbors as the wife of a traitor. Schmid was a true “bee”, while the army and the neighbors acted as “ants”. Human “bees”, just like the real bees pollinating the plants on our planet, are doing good for everybody. However, just like real bees, human “bees” frequently get little recognition for their essential contributions to the good of society. Worldly recognition goes to human “ants” and “leeches”. Just like real ants sacrifice their lives for their hive while fighting to the death with ants from competing hives, human “ants” are competitive workers who are well-embedded in their in-group and work hard to get ahead. It took the human ants in the German army over 50 years to change the moral code of their in-group and give Sergeant Schmid recognition for his ethical behavior by renaming a military base after him. While human ants value loyalty within their in-group, human “leeches” are egoists. Just like real leeches, which steal their victim’s blood for themselves, human “leeches” only care about their benefits with little regard for the welfare of others.

“Bees” are ethical, “ants” might have firm morals, while “leeches” are un-ethical. While colloquially the terms ethics and morality are frequently used interchangeably, many philosophers, going back to Aristotle and Spinoza^1,2^, see ethics as the standard for discerning “good vs. bad” or “right vs. wrong” based on societal values, while they associate morality with the personal attitude of individuals towards others^3^. This means that ethical people are universally good, in the sense of “universalists” as defined in the Theory of Basic Human Values by Schwartz et al.^4^. Moral people care for the welfare of members of their in-group while having limited tolerance for behavior that deviates from their norms^5^. For instance, even people who support gay marriage think that gay sex is immoral^6^. Morals thus define a personal value system. People who share similar morals aggregate in virtual tribes, such as pro or contra abortion, or pro or contra vaccines^7^. While social pressure gets human “bees”, “ants”, and “leeches” to claim to act by high ethical standards, their underlying value systems exhibit radical differences. Just pretending to be ethical does not make one ethical. Enron had the most beautifully written code of ethics, while its entire upper-level management definitively behaved highly unethical, following their own “moral code” of personal greed^8^. Applying the Schwartz system^9^ of personal values, a “bee” would be an ethical adherent of universalism and benevolence, understanding and protecting all people’s welfare and nature. “Ants” and “leeches”, on the other hand, are strongly motivated by self-enhancement, striving for achievement and power. The key difference between the two is that “ants” highly value tradition and loyalty to other members of their in-group, while “leeches” only care about their own interests, with no concern for the welfare of others. In other words, bees are “ethical”, ants are “moral”, and leeches are “amoral”. Differently from “bees”, “ants” and “leeches” will thus stick to the moral value systems of their in-groups which might be ethical or unethical, with little compassion for the rest of society.

In this paper, we explore the following question: how are ethical values correlated with individual and company performance? Research is contradictory, with some researchers finding that ethical leaders will create higher-performing organizations, while others find that unethical individuals will be promoted faster. Although religion and the law want individuals and companies to restrict competitive behavior and act ethically and according to social and community expectations, the reality is quite different. In business, law, and medicine, the concept of ethics serves as a personal code of conduct for people working in those fields, and ethical decisions themselves are often contested and challenged^10–12^. Frequently, “who breaks the law without being caught” wins. For instance, personality characteristics of psychopaths and CEOs show worrying similarities^13,14^. Frequently the most egotistical person is chosen as the leader of an organization^15^. On the other hand, ethical leaders are highly appreciated by their subordinates. While authoritative and inflexible leadership might have worked in an earlier era, today’s workers demand inclusiveness, empowerment, and a collaborative approach to problem-solving. Employees do not respond positively to top-down leadership, commonly considered outdated and counterproductive. Rather, they expect managers to follow humble, servant, and ethical leadership styles that are conducive to a work environment that enhances trust and builds positive relationships^16,17^.

Leaders in ethical organizations adopt collaborative approaches to promote engagement and fair behaviors without using authoritative power^18^. In traditional bureaucratic organizational models, leaders issue commands and expect compliance from subordinates, often through authoritative power. In organizations dominated by a command-and-control style, employees are not empowered to change a course of action even when they witness unethical or unlawful behaviors. Empirical evidence has shown how ethical leadership models enable followers to make decisions moving away from domineering or self-centered approaches^18^. Ethical and humble leadership has been associated with the perceived effectiveness of leaders, employees’ job satisfaction and dedication, and their willingness to report problems to management. Ethical leaders encourage normative behavior and discourage unethical behavior of their subordinates by being an ethical example, treating people fairly, and actively managing morality^19^.

Previous research has traditionally explored the association between ethical behaviors and outcomes by adopting qualitative methods, including surveys and self-report questionnaires. Our study contributes to this literature on ethical decision-making by providing a complementary methodology based on the digital traces that individuals leave as they interact online. In this study, we leverage the latest advances in natural language processing (NLP) and build “bee”, “ant”, and “leech” “tribes” of ethical, moral, and amoral people. Tribes are groups composed of members connected through a common belief or ideology. The concept has been used primarily in the marketing literature to describe consumer behavior^20^. Individuals in the same tribe share similar behaviors and similar ethical values and emotional responses to external stimuli^21^. In the rest of this paper, we will use the term “ethical values” as the goal to aspire to, distinguishing between ethical bees, moral ants, and amoral leeches.

## Ethical values, behaviors, and performance

The relationship between ethical values and behavior has attracted the interest of social scientists for several decades^22,23^. Values are defined as desirable goals that act as guiding principles in people’s lives. They are then translated and become visible through individual behaviors and concrete actions. Values may be important to some people and unimportant to others^24^. Ethical identity has been positively related to prosocial behaviors such as charitable giving and negatively related to unethical behaviors such as lying^25^. Ethical identity acts as a “self-regulatory mechanism” embedded in people’s internalized notions of right and wrong, influencing individual ethical behavior^26^. To help resolve important behavioral and ethical issues—including discrimination or sexual harassment—scholars have stressed the role of universal ethical values in defining corporate codes of ethics^9^.

The importance of ethical values in organizations is clearly explained by studies that document significant and positive relationships between firms’ social responsibility and financial performance^27^. Ethical decision-making and ethical leadership have been associated with increased business performance measured at the individual level. For instance, a case study of supervisor-subordinate dyadic data from Taiwanese organizations showed that subordinates’ business ethical values are positively associated with job performance and employee engagement^28^.

According to the social learning theory, individuals learn appropriate behaviors through a role-modeling process by observing the behaviors of others around them^29^. Studies show that team members exposed to similar cues regarding norms and ethical behaviors tend to behave homogeneously. Group norms are formed and reinforced by leaders’ behaviors, as they communicate as role models the importance of ethical values and use punishment and reward systems to encourage behaviors that align with cultural and universal values^30^. Empirical studies across various countries show that the ethical behavior of peers has the most significant impact on both individual moral values^31^ and group ethical behavior^32^. Ethical leaders will influence their subordinates to adjust their morals to be more ethical. A 2020 study on ethical leadership in business confirms that ethical values, especially when modeled by leaders, enhance both individual and business performance^33^.

A few studies have focused on measuring ethical values and ethical behaviors through the lenses of the big five factors of personality, suggesting that conscientiousness, agreeableness, and emotional stability are most consistently related to ethical leadership and agreeableness with power-sharing and fairness^34^. Recent empirical studies of European and African managers found that fairness of performance evaluation is associated with job satisfaction and mediated by trust and organizational commitment^35^. Other research has shown that satisfied employees increase business success^36^. In combination, this demonstrates that adhering to ethical values such as fairness will increase business performance. For example, research by Bowen et al.^37^ indicates that just and fair behaviors in the workplace translate into increased customer satisfaction. Other studies exploring the impact of organizational justice in HRM practice provide evidence that behaviors that “honor the justice principles” positively impact both job satisfaction and overall job performance^38^.

## Research design

Traditional approaches to measuring ethical values and ethical decision-making rely on data collected through surveys, questionnaires, or focus groups. For instance, a study involving middle-level managers and engineers at an aviation center relied on questionnaires to demonstrate the impact of ethical behavior on turnover intention^39^. Knafo and Sagiv^40^ conducted 603 phone interviews with Israeli families to explore the relationship between values and occupational environments. Schwartz et al.^41^ developed the Portrait Values Questionnaire (PVQ) based on Schwartz’s theory of values, which identified ten fundamental individual values influencing human actions^42^. However, the survey-based approach has considerable disadvantages, as individuals are notoriously bad at self-assessment, either seeing themselves in too positive a light or being overly critical of themselves. Researchers have repeatedly found that an individual’s friends are much better at rating the individual’s personality traits than the individual^43,44^. AI and machine learning put new tools at the disposal of behavioral and organizational researchers, allowing them to automatically analyze electronic traces of individuals to predict their personality characteristics. AI thus leverages the “wisdom of the swarm” to extend the judgment of friends by aggregating the assessment of large groups of people of the personality traits of an individual.

To overcome these limitations traditionally associated with survey methods^45^, we use a system called Tribefinder, which scans digital documents—including emails and social media posts—through a deep learning algorithm and considers the use of similar words in similar contexts. Tribefinder identifies tribal affiliations of individuals based on the words used by “tribal leaders”^46^. Tribefinder builds models of different tribes using LSTM^47^ and Tensorflow^48^ and trains their models with the Twitter streams of tribal leaders. The Machine Learning system built into Tribefinder assigns tribal membership based on word usage of individual tribe members on social media. The system proved to reach high classification accuracy values and Cohen’s Kappa^49^. It computes a dictionary of tribal words and their distribution in the text using a probabilistic distribution of a dictionary of millions of words called “word embeddings”. Once a tribe is created, the tribe members are plotted in proximity to each other, based on word usage and how they fit in with the predefined tribes^7,50,51^.

Researchers have been using machine learning to identify ethical tribe categories based on the content shared on Twitter^46^ or via email^21^. For instance, Morgan and Gloor^21^ analyzed the communication habits of three morality tribes, i.e., nerds, treehuggers, and fatherlanders, and found that these tribes significantly differ in how they communicate by email. Recent research has used digital traces such as emails and social media posts to predict emotional and behavioral traits from email communication. Gloor and Fronzetti Colladon^51^ found that communication patterns measured through e-mail interaction correspond with the ethical values of a person.

Motivated by the discussion on the impact of ethics on performance in the previous section, we explore ethical and unethical behavior via the words used by team members, categorizing individuals into three tribes, ethical “bees”, moral “ants”, and amoral “leeches”.

### The ethical tribes framework

To identify automatically tribal affiliation of “bees”, “ants”, and “leeches”, three tribes for Tribefinder were created, with the bee tribe leaders being open-source developers and artists, the ant tribe members being competitive athletes, and the leech tribe leaders being hedge fund managers and peddlers of “getting rich quick” schemes. In general, we relied on the procedure suggested by Gloor^52^, where AI-based methods are introduced to identify the personality, moral values, and ethics of individuals based on their body language and interaction with others. Additionally, six other “personality attribute tribes” were created to cross-verify the bees, ants, and leeches. We have chosen the representatives of these personality attribute tribes based on their perception in newspapers such as USA Today and People magazine and on Websites such as quora. Indeed, it has been shown that the language that individuals use in blogs and online forums can be a strong signal of their personality^53^. For instance, for the “arrogance” tribe, members were chosen from celebrities with a reputation for arrogance, such as Charlie Sheen or Will Smith. For the “modesty” tribe we chose celebrities with a reputation for modesty, such as the Dalai Lama and Emma Watson. For the “fairness” tribe we considered social advocates and human rights activists. Lastly, the “unfairness” tribe was built based on people like the editor of “Breitbart News” and hedge fund managers. The last two tribes are the “interest” tribe – subsuming curiosity, a passion for learning, and exploration of unknowns, with members such as Steven Pinker and Bill Gates—and a “disinterest” tribe of “couch potatoes”, that are individuals who are primarily interested in their hedonistic pleasures with members expressing their boredom on their Twitter profiles. It was quite hard to identify exemplary members for each tribe as, for instance, Lady Gaga has a reputation for being a comparatively modest down-to-earth artist, but artists in general by nature are gregarious extroverts and anything but modest. We, therefore, carefully cross-checked each member of these tribes by looking at their tweets and making sure that the tweets of members of the modesty tribe showed a very low arrogance score, which helped eliminate celebrities like Lady Gaga from the tribe.

### Data and performance metrics

To verify the validity of our approach, we carry out an email network and content analysis, considering three different e-mail archives. For each archive, we build a social network based on the email interaction of individuals and teams, and we analyze the content of email bodies or subject lines. In this network, each email account is represented as a node, with emails translating into one or more links connecting different nodes.

The first dataset, called “COINcourse”, consists of three cohorts of students enrolled in an international seminar on Collaborative Innovation Networks over three semesters, with a total of 89 students working in 21 groups. The performance metric is the final grade for each group, given by three instructors. The email archive consists of 89 students sending a total of 871 emails. The contents of emails sent by the 89 students were used to calculate their behavioral and emotional scores.

The second dataset, called “Healthcare Innovation”, consists of emails exchanged by 101 group members working in 11 innovation teams in the healthcare environment. The performance, innovation, and learning behaviors of each team were rated every other month for a year by three supervisors, who individually rated team performance, the capability of a team to learn new things, and the innovativeness of problem-solving methods.

The total email dataset includes 1782 actors (the outgroup) sending 286,029 emails, which was used for calculating the network metrics, while the content of the 191,519 emails sent by the 101 group members (the in-group) was used for calculating their behavioral and emotional scores.

The third dataset, called “Service Company”, consists of 91 managers who are part of 17 groups serving 17 large international customers of a global services firm. The managers are rated individually by their supervisors using three categories: outstanding, excellent, and good. The group performance is rated using the Net Promoter Score (NPS) collected from each group’s customers. NPS is a measure of customers’ loyalty to a company and is calculated using the answer to a key question “On a scale from 0 to 10, how likely is it that you would recommend a company (or brand) to a friend or colleague?”^54^. The total email dataset includes 1752 actors who sent 769,125 emails (the outgroup). This was used for calculating the network values, while the subject lines of the 126,978 emails sent by the 91 managers (the in-group) were used to calculate their behavioral and emotional scores. Note that, for this dataset, we were only able to obtain the subject line of emails, instead of the content of the entire email exchange, because of privacy restrictions. However, it has been shown in earlier work that for e-mail content analysis, metrics derived from the subject line are correlated with metrics derived from contents^55^.

Research has shown an intrinsic connection between ethical behavior and emotional response to an event. To support our method, we relied on the Basic Emotion theory, which proposes that human beings have a limited number of “biologically basic” emotions, including fear, anger, joy, and sadness^56^.

A different classification has been offered by the Dimensional Theory of emotion, which uses three dimensions: pleasant-unpleasant, tension-relaxation, and excitation-calm^57^, or various adaptations of the Circumplex model, where each emotion is located on a quadrant that reflects varying amounts of hedonic and arousal properties. Other researchers investigated the role of specific emotions, including shame and empathy, as they play a fundamental role in morality, with guilt being often considered the quintessential “moral emotion”^58^.

To improve the accuracy of the algorithm built within the Tribefinder, we chose the framework of the Basic Emotion Theory, as it proposes a basic classification of four fundamental emotions, namely fear, anger, joy, and sadness. These emotions have been preserved because of their biological and social functions are associated with an organized recurring pattern of behavioral components^59,60^. The Basic Emotion Theory was adopted by a recent study that used facial emotion recognition to predict emotional response to visual stimuli, which highlights the strong association between personality characteristics and moral values of individuals^61^. Based on an individual’s moral values, the individual will show different emotional responses. Therefore, besides the personality tribes, we also compute the emotionality of the emails using four categories: anger, fear, happiness, and sadness. The Tribefinder was trained to recognize these emotions, following a procedure similar to that used to classify personality attributes, i.e., training an AI model. We focus on these basic emotions as they have been considered by many to be the prototypical ones^62^. A combination of these emotions leads to more complex ones, as shown by Ekman’s Basic Emotions Theory^63^. The Basic Emotion Theory represented an appropriate framework to train the AI algorithm behind the Tribefinder, as it offers a classification of a limited number of emotions (i.e., fear, anger, joy, sadness) that are biologically and psychologically “basic” to all human beings.

In addition to analyzing the language used in email communication, we calculated key social network metrics, including degree centrality, betweenness centrality, and average response time^64,65^, to identify individual prominence (degree centrality) and information brokerage (betweenness centrality). The average response time (ART) indicates how fast an individual or a group responds to e-mails, offering insights into the degree of respect that an individual commands and the level of commitment they show^66^. We also distinguished between “alter ART” and “ego ART”, respectively indicating the time taken by recipients to answer an actor’s emails and the time taken by that actor to answer the emails they receive. These metrics are part of the six honest signals of collaboration described by Gloor^67^. Table 1 briefly summarizes the study variables.

**Table 1:** Description of the variables.

	Description of variables	Calculation and source
**Independent variables**		
Behaviors	Behavioral scores Fairness vs unfairness Arrogance vs modesty Interest vs disinterest	Application of the Tribefinder classifications to Email archives
**Control variables**		
Emotions	Emotional scores Anger Fear Happiness Sadness	Application of the Tribefinder classifications to Email archives
**Control variables**		
Network dynamics	Social network position Degree centrality Betweenness centrality Average response time	Using in-group Email network Total number of other nodes to which a node is adjacent Total number of shortest paths between every possible pair of nodes that go through a given node Average time that it takes a person to reply to an Email
**Dependent variable**		
Performance	Dataset #1—COIN course Group performance	Final grade
	Dataset #2—Healthcare Innovation Group performance	Bi-monthly rating by leadership (learning, performance, innovation)
	Dataset #3—Service Company Individual performance Group performance	Assessed by supervisors Evaluated by clients and measured through NPS

## Results

Our analysis followed two steps. First, we examined the behavior of people classified as bees, ants, and leeches and then related these roles to performance with metrics at both the individual and group level.

### Behavioral trends for bees, ants, and leeches

**Figure 1:**
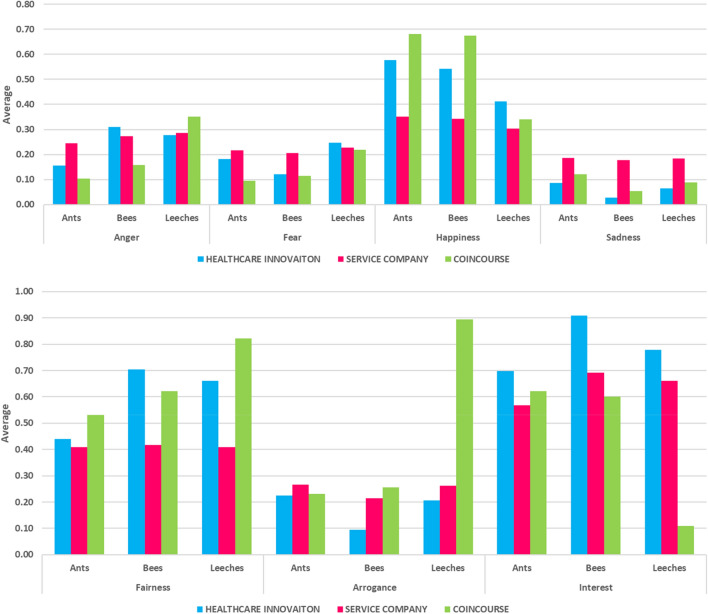
Average emotional and behavioral scores of ants, leeches and bees.

Figure 1 shows the average values for both emotional (i.e., anger, fear, happiness, and sadness) and behavioral scores (i.e., arrogance, fairness, interest) of ants, leeches, and bees—while considering the three datasets described in the previous section.

To evaluate the significance of mean differences, we carried out an analysis of variance, as presented in Tables 2, 3 and 4. Instead of using a classic ANOVA, we used Welch’s ANOVA as a robust alternative in the case of unequal group variances—as indicated by the results of the Levene’s tests that we performed for all groups. Accordingly, we also ran a robust post-hoc analysis to evaluate significant group differences through Games-Howell tests.

**Table 2:** Welch’s ANOVA—COINcourse dataset.

Variable	Welch’s ANOVA significance	Games–Howell post-hoc tests			
Anger	0.000		Ants	Leeches	Bees
		Ants		***	
		Leeches	***		***
		Bees		***	
Fear	0.000		Ants	Leeches	Bees
		Ants		**	
		Leeches	**		***
		Bees		***	
Happiness	0.000		Ants	Leeches	Bees
		Ants		**	
		Leeches	**		***
		Bees		***	
Sadness	0.056		Ants	Leeches	Bees
		Ants			
		Leeches			^
		Bees		^	
Fairness	0.001		Ants	Leeches	Bees
		Ants		^	
		Leeches	^		***
		Bees		***	
Arrogance	0.000		Ants	Leeches	Bees
		Ants		***	
		Leeches	***		***
		Bees		***	
Interest	0.000		Ants	Leeches	Bees
		Ants		***	
		Leeches	***		***
		Bees		***	

^p < 0.1; *p < 0.05; **p < 0.01; ***p < 0.001.

**Table 3:** Welch’s ANOVA—Service Company dataset.

Variable	Welch’s ANOVA significance	Games–Howell post-hoc tests			
Anger	0.007		Ants	Leeches	Bees
		Ants		**	
		Leeches	**		
		Bees			
Fear	0.050		Ants	Leeches	Bees
		Ants			
		Leeches			^
		Bees		^	
Happiness	0.000		Ants	Leeches	Bees
		Ants		**	
		Leeches	**		*
		Bees		*	
Sadness	0.794		Ants	Leeches	Bees
		Ants			
		Leeches			
		Bees			
Fairness	0.872		Ants	Leeches	Bees
		Ants			
		Leeches			
		Bees			
Arrogance	0.000		Ants	Leeches	Bees
		Ants			*
		Leeches			***
		Bees	*	***	
Interest	0.000		Ants	Leeches	Bees
		Ants		***	***
		Leeches	***		^
		Bees	***	^	

^p < 0.1; *p < 0.05; **p < 0.01; ***p < 0.001.

**Table 4:** Welch’s ANOVA—Healthcare Innovation dataset.

Variable	Welch’s ANOVA significance	Games–Howell post-hoc tests			
Anger	0.000		Ants	Leeches	Bees
		Ants		**	***
		Leeches	**		
		Bees	***		
Fear	0.000		Ants	Leeches	Bees
		Ants			
		Leeches			***
		Bees		***	
Happiness	0.000		Ants	Leeches	Bees
		Ants		**	
		Leeches	**		***
		Bees		***	
Sadness	0.000		Ants	Leeches	Bees
		Ants			*
		Leeches			**
		Bees	*	**	
Fairness	0.000		Ants	Leeches	Bees
		Ants		***	***
		Leeches	***		
		Bees	***		
Arrogance	0.000		Ants	Leeches	Bees
		Ants			**
		Leeches			***
		Bees	**	***	
Interest	0.000		Ants	Leeches	Bees
		Ants			***
		Leeches			***
		Bees	***	***	

^p < 0.1; *p < 0.05; **p < 0.01; ***p < 0.001.

As shown in Fig. 1 and Tables 2, 3 and 4, bees and ants are less arrogant than leeches. Bees are also more interested and less fearful than leeches. Surprisingly, ants seem to be the happiest group. Our post-hoc analysis reveals that the most significant differences are usually between ants and leeches and between bees and leeches. This is partially dependent on the datasets used in the study. For example, significant differences between ants and bees emerge in the Healthcare Innovation dataset.

The analysis of social network metrics indicates the presence of different behavioral patterns, again depending on the dataset. For example, we find that bees are much more central in the email network while considering the COINcourse and Healthcare Innovation dataset—both in terms of degree and betweenness centrality. On the other hand, leeches are more active—they send more messages and have a higher degree—in the Service Company dataset.

### Performance of bees, ants, and leeches

As the second step of the analysis, we looked for a relationship between performance and the individual classification of participants as ant, leech, or bee. The regression analysis produced the models presented in Tables 5, 6 and 7, which show the best models for each dataset. All our models were tested to exclude multicollinearity problems. The Variance Inflation Factor (VIF) values were reasonably low—always lower than 2.5 and, in most cases, also lower than 2.

**Table 5:** Regression analysis—Service Company dataset.

Variable	Individual performance	Group performance
Ant dummy	0.825**	
Arrogance	− 0.898*	
Average interest		− 3.685*
Average degree		− 0.003*
Number of leeches		− 0.096^
Average arrogance		− 3.570^
Average ego ART		− 0.071**
Constant	1.438***	6.274**
Adjusted R^2^	0.139	0.437
N	87	17

^p < 0.1; *p < 0.05; **p < 0.01; ***p < 0.001.

**Table 6:** Regression analysis—Healthcare Innovation dataset.

Variable	Group performance	Group innovation	Group learning
Number of bees	0.738*		0.683*
Number of leeches		− 86.181*	− 10.581*
Average degree		− 0.125*	
Average happiness		754.071**	
Average fear		1423.264^	
Average fairness			− 35.550*
Average arrogance			61.533*
Constant	17.051***	− 257.007	28.341*
Adjusted R^2^	0.444	0.623	0.715
N	11	11	11

^p < 0.1; *p < 0.05; **p < 0.01; ***p < 0.001.

**Table 7:** Regression analysis—COINcourse dataset.

Variable	Group final grade
Number of bees	0.151*
Number of leeches	− 0.167*
Average arrogance	0.898**
Average betweenness	0.005*
Constant	1.040***
Adjusted R^2^	0.450
N	21

^p < 0.1; *p < 0.05; **p < 0.01; ***p < 0.001.

In Table 5, we present the effect of the three categories (ants, bees, and leeches) on the group and individual performance, only relating to the Service Company dataset.

As already mentioned, individual performance was judged by the supervisors of the managers participating in the study, while group performance was evaluated by the company’s clients and measured as customer satisfaction through the NPS indicator.

Results from the regression analysis (Table 5) indicate that individual ratings are higher when managers are less arrogant and in the ant category. On the other hand, more variables contribute to group performance, i.e., client satisfaction. Groups that received higher evaluations answered emails faster, had a lower number of leeches, and comprised less arrogant employees. Employees in these groups were also characterized by a lower degree centrality and lower interest. In other words, these employees were more focused on a smaller number of key customers, to whom they gave preferential treatment by answering them more quickly and talking less about topics of general interest.

In Table 6, we present the analysis carried out on the Healthcare Innovation dataset, where 11 groups were evaluated with respect to performance, innovation, and learning skills.

As Table 6 shows, the presence of bees is particularly relevant for a good group performance. For innovation tasks, on the other hand, it seems more important to have focused communication (having a lower degree) and as few leeches as possible. Groups that present high innovation skills are more emotional, exhibiting higher levels of happiness and fear. Lastly, the presence of bees (and a low number of leeches) seems to favor group learning. Surprisingly, learning abilities are also higher when group members are less fair and more arrogant.

Table 7 shows the best regression models for the COINcourse dataset, where a group of teachers evaluated 21 groups of students. Grades had continuous values, ranging from 1 to 2—with 2 representing the highest grade and 1 the lowest.

In the COINcourse dataset, student groups that achieved a higher grade had more bees and fewer leeches (see Table 7)—which is aligned with the results obtained for Group Learning in the Healthcare Innovation dataset. In addition, it seems that having higher betweenness centrality (probably increasing the possibility of integrating knowledge coming from multiple sources) is beneficial to performance. Surprisingly, groups with higher average levels of arrogance achieved a higher grade. This might have to do with the students’ self-esteem, in that groups that were more self-assured in their presentations got a higher grade from their instructors.

## Discussion

The analysis of variance across the three datasets suggests that the most significant differences in terms of emotional and behavioral scores are between ants and leeches and between bees and leeches. Regardless of the datasets and related industries (healthcare, higher education, or service companies), leeches are systematically emerging as being more arrogant and with less curiosity and passion for learning than bees. Leeches are also the tribe with the lowest happiness levels. This is consistent with previous studies looking at narcissistic behaviors through the lenses of social media posting. For example, leeches in our study display partially similar traits to the “takers”, a personality type described by Adam Grant^68^: these individuals tend to be more self-promoting, arrogant, boastful, prone to anger, and self-absorbed.

Our study also found that bees are less fearful than leeches. A possible explanation is that bees are driven by collaborative values of helping others independently of what they can receive in exchange. An interesting result is that ants seem to be happier than leeches. This might be explained by their desire to conform to society and rely on social norms to feel accepted by other members of their organization. Ants may be happier because their behavior better aligns with the social norms of their community, making them feel cheerier and at ease in their community. As demonstrated by Helliwell^69^, people tend to be happier when they work together for a worthy, non-individualistic purpose.

Results were not always consistent across datasets. In the service company, individual performance was higher when managers were less arrogant and displayed traits typical of the “ant” tribe, such as valuing conformity and security and being tendentially conscientious and fair. This can be explained by a tendency of managers to provide positive assessments to employees who “fit the mold”, who are more aligned with expectations and follow shared values and morals. Since “bees” tend to take more social risks and are open to trying new things, this could translate into less easy behaviors to manage and control. Not surprisingly, groups that received higher evaluations were the ones that answered emails faster. This is aligned with previous studies showing that responding to emails at a reasonably fast rate improves customer satisfaction^70^. The highest performing groups also had a lower number of leeches and were composed of fewer arrogant employees, which is consistent with previous studies demonstrating the importance of humility and its impact on performance^71^. For instance, Nevicka et al.^72^ found that a leader’s narcissism inhibits information exchange between group members, negatively impacting group performance.

In the Healthcare Innovation dataset, the presence of bees—i.e., individuals who are open to learning, try new things, and care for others—has a positive impact on group performance. A possible explanation is that bees might be acting as motivators for the group promoting idea generation, thanks to their tendency to embrace social risks and be open to new things. Results from the healthcare innovation dataset indicate that having leeches in your group may decrease your collective ability to innovate and learn. This might be explained by the tendency of leeches to favor self-promotion and to advertise their accomplishments rather than advancing the group’s goal^73^. Groups focused on innovation tasks might benefit from having a lower degree centrality, reducing the number of connections to others, and inviting as few leeches as possible. As demonstrated by Buffardi and Cambell^73^, people who behave like leeches have significantly more friends, often establishing superficial connections with the mere goal of advertising themselves. Because innovation, in particular at the idea generation stage, is a process of trial and error that leads to fluctuating emotions, it was not surprising to see that highly innovative groups were more emotional. Throughout the creative process of idea generation, it is natural to go through happiness when groups push things forward and emotions can turn negative when new problems emerge. Experiencing fear is also a possible sign of commitment, as group members demonstrate attachment to the project and care about the group success. While negative emotions might sometimes act as distractors in the professional and personal sphere, discrete negative emotions like fear and anger may spark proactivity and can be expected during the chaotic innovation process, as shown by studies exploring the relationship between affect and creativity^74^. Another result from the Healthcare dataset—consistent with the positive effect of bees on group performance—is that groups improve their learning outcome if there are more bees and fewer leeches among them. Again, bees are characterized by a desire for learning and caring for others which are roles often associated with open innovation^75^. The surprising result that learning abilities are higher when group members are less fair and more arrogant might be explained by the need to incorporate some level of competitiveness within and among groups in order for learning to occur. See Cagiltay et al.^76^ for a detailed explanation of how competition enhances learning and motivation.

The results of the analysis conducted on the COINcourse dataset confirm the same conclusion we found in the Health Innovation dataset regarding the importance of forming groups that have more bees and fewer leeches. In this case, groups increased their chance of getting a good grade if there were more caring students and fewer self-centered students. At the same time, a surprising result was that when groups acted more arrogantly, they had a higher chance to get a higher grade, which contrasts with other studies showing that arrogance is negatively related to self-and other-rated task performance^77^. The impact of arrogance on group performance might be explained by incorporating the natural level of competitiveness that students develop prior to any class presentation. Higher group performance was also associated with higher betweenness centrality, highlighting the important role of students who acted as spokesperson (i.e., team leaders) and knowledge brokers between group members, other groups, and the instructors.

**Figure 2:**
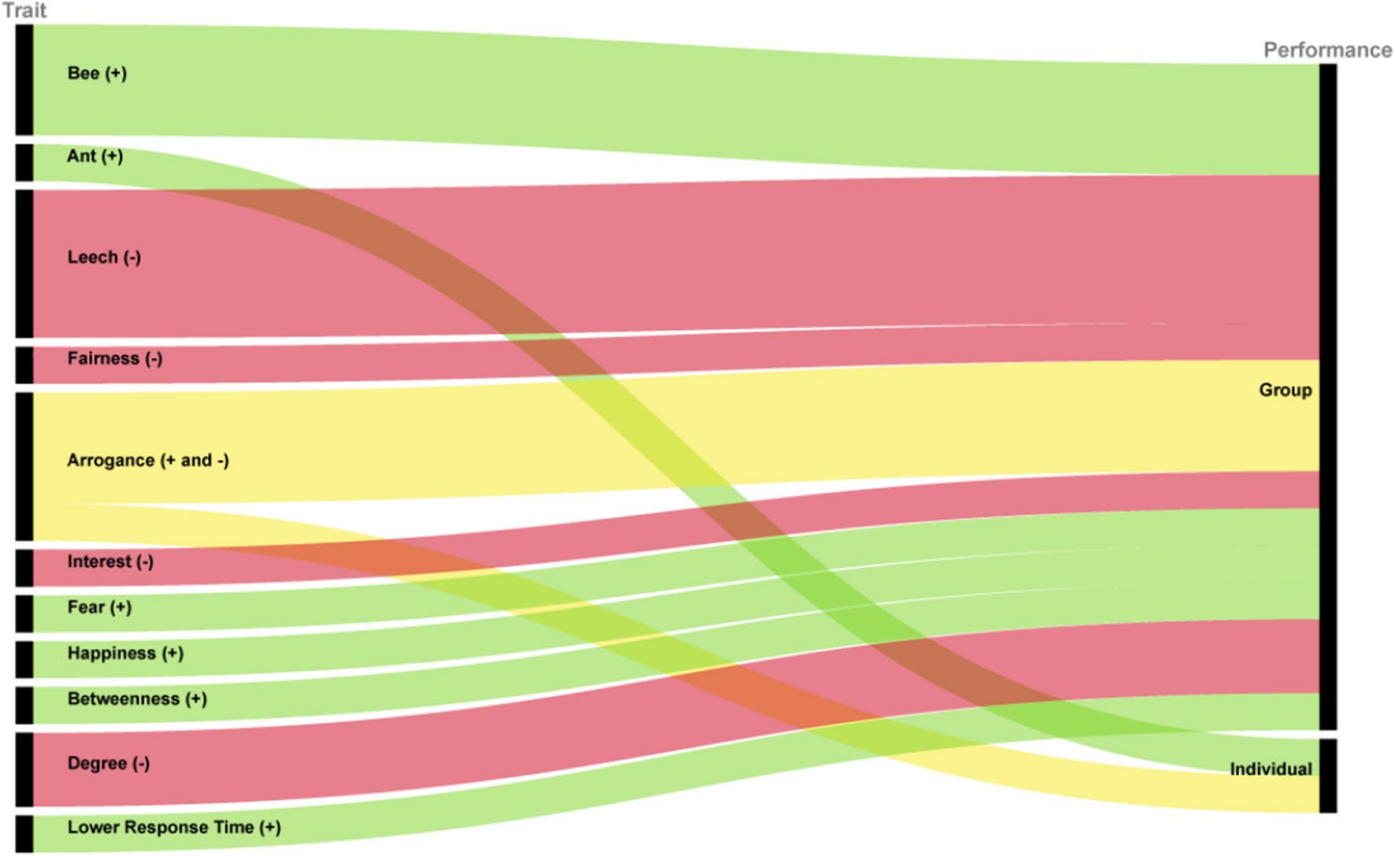
Variables impacting group and individual performance.

Figure 2 summarizes the number of significant relationships we find in our best regression models, linking individual and group performance with individual traits and social network dynamics.

## Conclusions and limitations

In this study, we investigated how organizations can effectively manage individual and team ethical behaviors—exemplified by bees and ants—while avoiding unethical ones—as exemplified by leeches. To this purpose, we measured the impact of behavioral and emotional traits on group performance, with a focus on the role of ethical behaviors in determining real-world success. Based on three different contexts, our findings indicate that exhibiting moral values, being fair, being open to others and new things, and caring for others, correlate positively or negatively with success depending on the tasks.

This study corroborates evidence from organizational psychology^68^ and ethical leadership^17^ by suggesting that building groups composed of individuals with traits and values typical of bees and ants can lead to success. In contexts where groups are asked to come up with innovative ideas and learn from others, the presence of arrogant leeches might be detrimental to success. In other contexts where groups are tasked with responding to customer questions and solving their issues, or when they are tasked with presenting results in a classroom environment, group performance is higher if individuals are more self-oriented, unfair, and take ethical risks. This is aligned with evidence provided by evolutionary biology studies, where social animals face recurrent opportunities to engage in nonzero-sum exchanges: humans and other mammals who engage in cheating rather than cooperative behaviors and react with emotions that induce them to play “tit for tat” have been found to have an advantage over those who had to figure out their next move using their general intelligence^23^.

While bees, ants, and leeches represent three fundamental styles of social interaction based on moral and behavioral values, the lines between them are not hard and fast. For example, having ants might be better for some tasks and detrimental for others, and different configurations might be better, depending on contexts and business cases.

This study contributes to the theories and practice of ethical decision-making by proposing the adoption of a new methodology based on computational social science that links ethical behaviors with business outcomes. The limited sample of individuals represents the main limitation of the study. Future studies should consider larger datasets and incorporate additional control variables, such as age, gender, or tenure within the organization, which we could not consider in this study due to privacy agreements. To conclude, we hope that in today’s age of big data, aggregating the ethical understanding of large groups of people through machine learning will assist in recognizing and rewarding the ethical courage of today’s “Anton Schmid” without a 50-year delay.

### Institutional review board statement

The study was conducted according to the guidelines of the Declaration of Helsinki and approved by the Institutional Review Board of MIT (protocol code 170181783) on 16 February 2017.

### Informed consent statement

Informed consent was obtained from all subjects involved in the study.

## Data Availability

The datasets generated during and/or analyzed during the current study are not publicly available due to privacy agreements.
